# Wall Shear Stress (WSS) Analysis in Atherosclerosis in Partial Ligated Apolipoprotein E Knockout Mouse Model through Computational Fluid Dynamics (CFD)

**DOI:** 10.3390/ijms25189877

**Published:** 2024-09-12

**Authors:** Minju Cho, Joon Seup Hwang, Kyeong Ryeol Kim, Jun Ki Kim

**Affiliations:** 1 Department of Convergence Medicine, Brain Korea 21 Project, College of Medicine, University of Ulsan, Seoul 05505, Republic of Korea; 2 Biomedical Engineering Research Center, Asan Institute for Life Science, Asan Medical Center, Seoul 05505, Republic of Korea

**Keywords:** atherosclerosis, plaque formation, computational fluid dynamics (CFD), wall shear stress (WSS), ApoE-KO mice, standard deviation

## Abstract

Atherosclerosis involves an inflammatory response due to plaque formation within the arteries, which can lead to ischemic stroke and heart disease. It is one of the leading causes of death worldwide, with various contributing factors such as hyperlipidemia, hypertension, obesity, diabetes, and smoking. Wall shear stress (WSS) is also known as a contributing factor of the formation of atherosclerotic plaques. Since the causes of atherosclerosis cannot be attributed to a single factor, clearly understanding the mechanisms and causes of its occurrence is crucial for preventing the disease and developing effective treatment strategies. To better understand atherosclerosis and define the correlation between various contributing factors, computational fluid dynamics (CFD) analysis is primarily used. CFD simulates WSS, the frictional force caused by blood flow on the vessel wall with various hemodynamic changes. Using apolipoprotein E knockout (ApoE-KO) mice subjected to partial ligation and a high-fat diet at 1-week, 2-week, and 4-week intervals as an atherosclerosis model, CFD analysis was conducted along with the reconstruction of carotid artery blood flow via magnetic resonance imaging (MRI) and compared to the inflammatory factors and pathological staining. In this experiment, a comparative analysis of the effects of high WSS and low WSS was conducted by comparing the standard deviation of time-averaged wall shear stress (TAWSS) at each point within the vessel wall. As a novel approach, the standard deviation of TAWSS within the vessel was analyzed with the staining results and pathological features. Since the onset of atherosclerosis cannot be explained by a single factor, the aim was to find the correlation between the thickness of atherosclerotic plaques and inflammatory factors through standard deviation analysis. As a result, the gap between low WSS and high WSS widened as the interval between weeks in the atherosclerosis mouse model increased. This finding not only linked the occurrence of atherosclerosis to WSS differences but also provided a connection to the causes of vulnerable plaques.

## 1. Introduction

Atherosclerosis is a systemic disease characterized by the formation of plaque within the inner lining of arteries, leading to inflammation. The accumulation of fatty material in the intima, the inner layer of the artery, leads to the formation of atherosclerotic plaque, which disrupts blood flow and causes thrombus and tissue ischemia [1]. This condition can result in ischemic stroke and heart disease, which remain the leading causes of adult mortality worldwide [2]. Numerous risk factors contribute to the development of atherosclerosis, including high cholesterol, hypertension, obesity, and diabetes [1]. Low WSS is another significant risk factor that promotes the formation and progression of plaque in the arteries due to lipid accumulation, while high WSS can cause damage to the vascular intima and inflammatory reactions that can contribute to the development of vulnerable plaque [3]. Vulnerable plaque is where a fibrous cap with inflammatory onset exists within a lipid rich core [4]. Thus, WSS is associated with the genetic expression of inflammatory biomarkers such as vascular cell adhesion molecule-1(VCAM-1), nuclear factor kappa-light-chain-enhancer of activated B cells (NF-κB), cluster of differentiation 31 (CD31), alpha-smooth muscle actin (α-SMA), etc. [3]. Since hemodynamic factors such as WSS and bio-inflammatory factors, along with systemic risk factors such as high-fat diet or obesity, are linked to progressive arterial injury and subsequent atherosclerosis, understanding these risk factors and their roles in the development of atherosclerosis is crucial for developing prevention and treatment [5,6,7].

CFD is a powerful tool for analyzing fluid flow, such as blood flow in this case [8]. CFD simulations enable the prediction of vascular diseases by analyzing aspects of blood flow and vascular geometry that are difficult to replicate in vivo [9]. Different from angiography or computed tomography, CFD analysis can identify hemodynamic changes before the formation of plaque. This method allows for predicting the progression of plaque using hemodynamic parameters [10]. WSS refers to the frictional stress exerted on the vessel wall. When frictional stress is lowered, substances such as lipid and cholesterol can accumulate on the inner wall of blood vessels, which is associated with the onset of plaque formation [6]. This experiment specifically employed the Bird–Carreau model, which uses non-Newtonian flow behavior in calculations [11].

**Figure 1:**
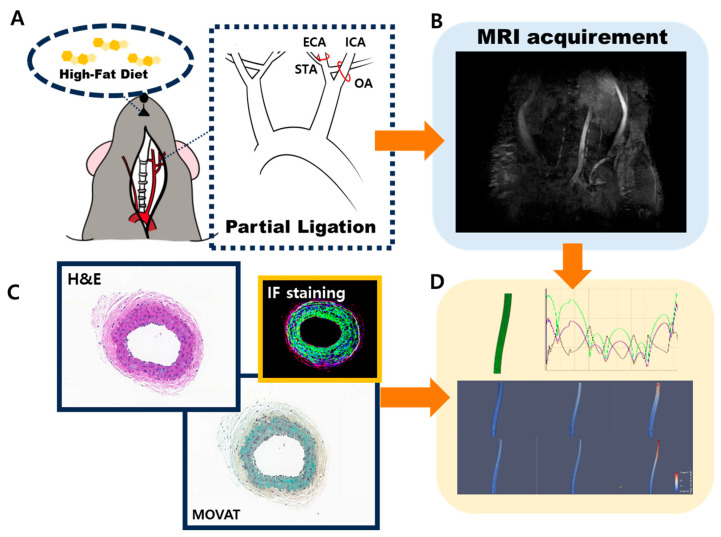
Scheme of WSS evaluation acquisition of ApoE-KO mice atherosclerosis model. (**A**) Partially ligated ApoE-KO mice fed a high-fat diet were developed as an atherosclerosis model. To confirm disrupted blood flow, (**B**) MRI images were acquired, along with (**C**) H&E, Movat staining, and IF staining images. Based on these results, (**D**) a 3D reconstruction of the carotid artery was performed to evaluate WSS.

In this study, ApoE-KO mice were used as an in vivo animal model of the formation of atherosclerosis. The lack of apolipoprotein E leads to the accumulation of cholesterol in the bloodstream and results in low lipoprotein clearance [12]. Since the disruption of blood flow and oscillatory WSS are associated with the formation of atherosclerosis, partial ligation of the left carotid artery (LCA) was applied to the mice to induce inflammation and block blood flow [13]. Additionally, the mice were fed a high-fat diet to promote acute plaque formation [14]. The blood flow was measured through MRI to confirm the disruption occurrence on the partially ligated LCA (Figure 1A–C). To observe the relationships between high cholesterol in the bloodstream, the increased expression of inflammatory factors, and the thickness of the intima at the bifurcation and middle part of the LCA with WSS, CFD analysis (Figure 1D) was conducted [8]. ApoE-KO mice were divided into three groups for 1 week, 2 weeks, and 4 weeks. The mice were examined to analyze WSS through CFD and biological factors that influence the development of atherosclerosis. Along with WSS, inflammatory bio-factors and pathological characteristics were identified to establish their relationship in atherosclerosis. Through CFD, an increase in WSS was observed, along with the dispersion and widening gap between low and high WSS over weeks. An increase in inflammatory biomarkers and the thickness of atherosclerotic plaques was also confirmed, establishing a correlation. Since the development of atherosclerosis is influenced by multiple factors, the goal was to simulate and visualize WSS within the vessels and align it with pathological causal relationships. Therefore, as a novel approach, the relative differences in shear stress within the vessel wall were compared to examine factors contributing to the development of atherosclerosis, such as vessel wall thickness and inflammatory substances, at different time intervals.

## 2. Results

### 2.1. Assessment of Atherosclerosis Formation via MRI and Histological Staining

**Figure 2:**
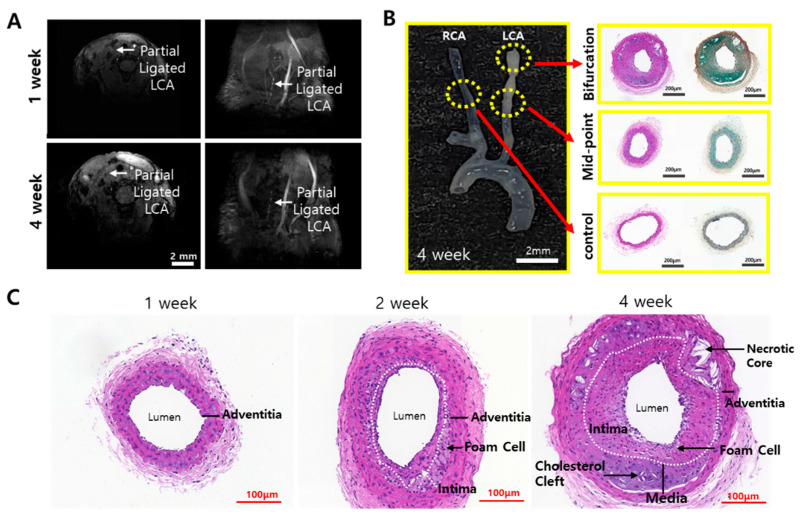
Blood flow disrupt confirmation through MRI TOF mode and histological staining of carotid artery. (**A**) Cross-sectioned MRI image and lateral MRI image of 1- and 4-week partial ligated ApoE-KO mice. Partial ligated LCA are indicated as white arrows in MRI images. (**B**) The excised sample of 4-week mice and H&E and MOVAT staining images of the cross-sectioned tissue at the partial ligation site (bifurcation), the midpoint, and the RCA without partial ligation as control. (**C**) H&E staining results of 1-, 2-, and 4-week LCA. Pathological features are marked in the image.

A partially ligated LCA disrupts the blood flow, and it can be confirmed by MRI. MRI is a non-invasive imaging technique that uses magnetic field gradients to obtain signals based on blood flow velocity [15]. In this experiment, the TOF mode was applied for blood flow measurement. TOF is commonly used in angiology to visualize blood vessels and blood flow dynamics without the use of contrast agents. Since a magnetic gradient produces a stronger signal in blood fluid than in the surrounding tissue, blood vessels can be easily visualized. To confirm the disruption of blood flow, partially ligated ApoE-KO mice with a high-fat diet underwent MRI imaging in the TOF mode. The mice were observed on a weekly basis. In the 1-week group, blood flow was observed in the LCA. However, in the 4-week group, a longer period of partial ligation and high-fat diet than the 1-week group contributed to the progression of atherosclerotic plaques, reducing blood flow, which was confirmed by MRI. The bloodstream became increasingly obstructed as the duration of ligation and high-fat diet in the mice lengthened, especially when comparing the mice from the 1-week group to those from the 4-week group, the blood disruption is shown to be significantly different (Figure 2A). This can probably be attributed not only to the disruption of blood flow caused by ligation but also to changes in blood flow due to the progression of atherosclerotic plaque. After MRI imaging, the mice were sacrificed, and then histological staining of the carotid artery samples was performed.

For H&E and MOVAT staining, plaque formation was observed in areas pathologically predicted to have lesions, as shown in Figure 2B. Atherosclerotic characteristics such as necrotic cores and foam cells appeared in the pathological staining, since foam cells are a major source of necrotic cores in the atherosclerosis plaque [16,17]. Figure 2C shows the 1-, 2-, and 4-week H&E staining results. Pathological features of plaque were not observed in the 1-week group mice samples. However, in the 2-week and 4-week group mice samples, the intima thickened, and foam cells began to appear. In the 4-week group samples, necrotic cores, along with foam cells and cholesterol clefts, were observed. Additionally, the intima appeared to be significantly thickened.

### 2.2. Immunofluorescence Staining Evaluation for Inflammation Marker

**Figure 3:**
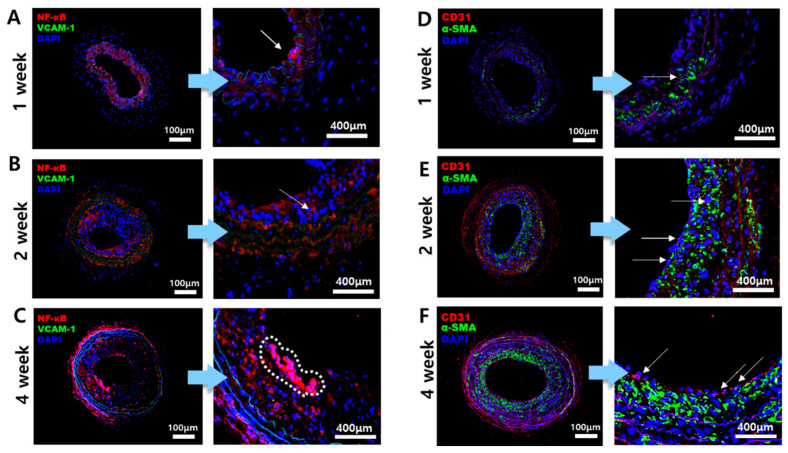
IF performed on the bifurcation part of the LCA. The samples were double-stained with VCAM-1 and NF-κB as one set, and CD31 and α-SMA as another set. (**A**–**C**) represent the 1-, 2-, and 4-week IF staining results for VCAM-1 (green) and NF-κB (red) in the LCA bifurcation. (**D**–**F**) show the staining results for CD31 (red) and α-SMA (green) in the LCA bifurcation, respectively. The arrows and dotted lines in the magnified images of each figure indicate the fluorescence biomarker. The arrows in Figures (**A**–**C**) represent NF-κB (red). The arrows in the magnified images of Figures (**D**–**F**) represent CD31 (red). For VCAM-1 (green), no significant observations were made, and α-SMA (green) was well visualized in Figures (**D**–**F**).

As plaque formation begins, the gene expression of inflammatory bio-factors increases, and it can be visualized through IF staining with target antibodies for the biomarkers. VCAM-1, NF-κB, CD31, and α-SMA were chosen for this experiment. VCAM-1 is one of the crucial molecules that contribute to the formation of atherosclerosis, becoming activated on the surface of arterial endothelial cells [18]. NF-κB is a transcription factor that shows increased expression when stimulated by low WSS on the inner side of the vessel [19]. NF-κB is also involved in general inflammatory responses [20]. CD31, known as platelet endothelial cell adhesion molecule-1(PECAM-1), expresses on the surface of endothelial cells, acting as a signaling receptor and stimulating the expression of inflammatory molecules on atherosclerosis lesions [21]. In addition, smooth muscle cells (SMCs) proliferate during the formation of atherosclerotic plaque, leading to intimal thickening and a change in their phenotype [22]. Since α-SMA is expressed in SMCs, atherosclerotic lesions should contain α-SMA as the intima thickens. As IF staining was conducted, the results are shown in Figure 3. The expression of NF-κB (red) increased in the 4-week mice group compared to the 1-week and 2-week mice groups, as shown in Figure 3A–C with an arrow indication. As the plaque thickened, the inflammatory response was activated, which can be confirmed by the expression level of NF-κB (Figure 3C). However, there was no significant difference in the expression levels of VCAM-1 (green). In Figure 3D–F, the expression of CD31 (red) is shown on the inner side of the intima in endothelial cells in the 4-week mice LCA while CD31 is less shown in the 1- and 2-week mice LCA. On the other hand, α-SMA (green) is shown in all 1-, 2-, and 4-week mice models, and the expression of α-SMA tends to be concentrated towards the intima, where plaque is formed. Through IF staining, it is confirmed that the inflammatory response occurred around atherosclerosis plaque. Additionally, it was confirmed that mice subjected to prolonged partial ligation and a high-fat diet had thicker plaques and atherosclerotic lesions, which corresponded with an increased expression of biomarkers.

### 2.3. Wall Shear Stress Acquirement

**Figure 4:**
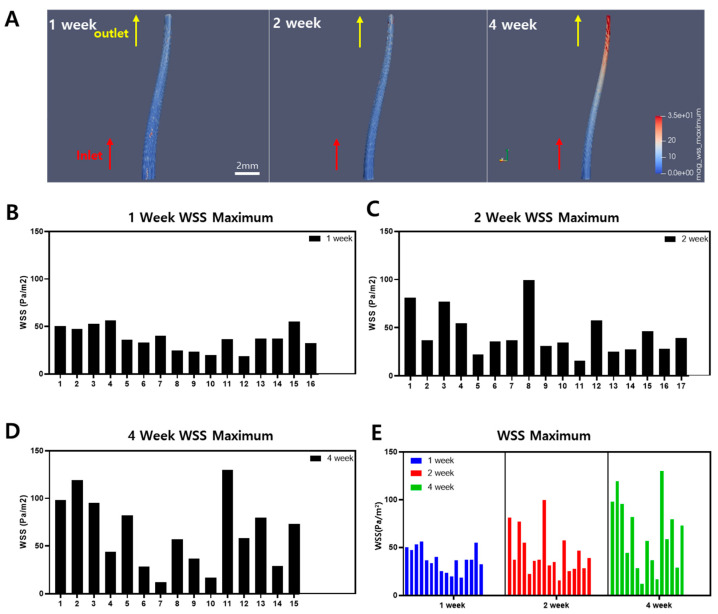
A 3D construction of the left carotid artery from the midpoint of LCA to bifurcation of the partial ligation part with WSS evaluation. (**A**) 3D image of LCA with WSS distribution; the red part refers to high WSS, where the blue part refers to low WSS. (**B**–**E**) are maximum WSS graphs for 1-, 2-, and 4-week mice in all groups, respectively.

To reconstruct the blood vessels, cross-sectional images of the ligated carotid artery and vessel images obtained from MRI were used to model the 3D structure of the ligated left carotid artery. The 3D images of the blood vessels were reconstructed to match the appearance of atherosclerotic plaques observed at 1-week, 2-week, and 4-week intervals in mice. This reconstruction reflected the narrowed internal diameter of the atherosclerotic arteries, allowing for CFD analysis. The blood flow inlet was set at the midpoint of LACE, and the outlet was set at the bifurcation. In Figure 4A, the blood flow was applied to the 3D remodeled LCA to measure WSS. The results of the blood flow simulation using fluid dynamics showed that WSS was higher at the bifurcation in the ligated mice at 1-week, 2-week, and 4-week intervals. As shown in Figure 4A, the areas with high WSS are shown in red, and in the mouse model with partial ligation and a high-fat diet for about 4 weeks, the WSS was relatively higher at the bifurcation compared to the midpoint of the vessel. Furthermore, in the 2-week mouse model, WSS also increased compared to the 1-week model. This indicates that the areas where atherosclerotic plaques are expected to form show higher WSS depending on the duration of the experiment. However, atherosclerosis formation is dependent on the low WSS [3,23].

The maximum WSS within the blood vessels of the 1-, 2-, and 4-week mice groups are shown in Figure 4B–D. The general tendency of WSS increases by the duration of the experiment regardless of some mice in the 1-week group showing high WSS and some mice in the 4-week group showing low WSS. This tendency is summarized in Figure 4E, where the maximum WSS of all groups of mice is presented in the graph. Since these results differ from the existing findings that atherosclerosis is caused by low WSS, TAWSS was compared for further analysis [24].

### 2.4. The Correlation between Wall Shear Stress and Atherosclerotic Lesions

**Figure 5:**
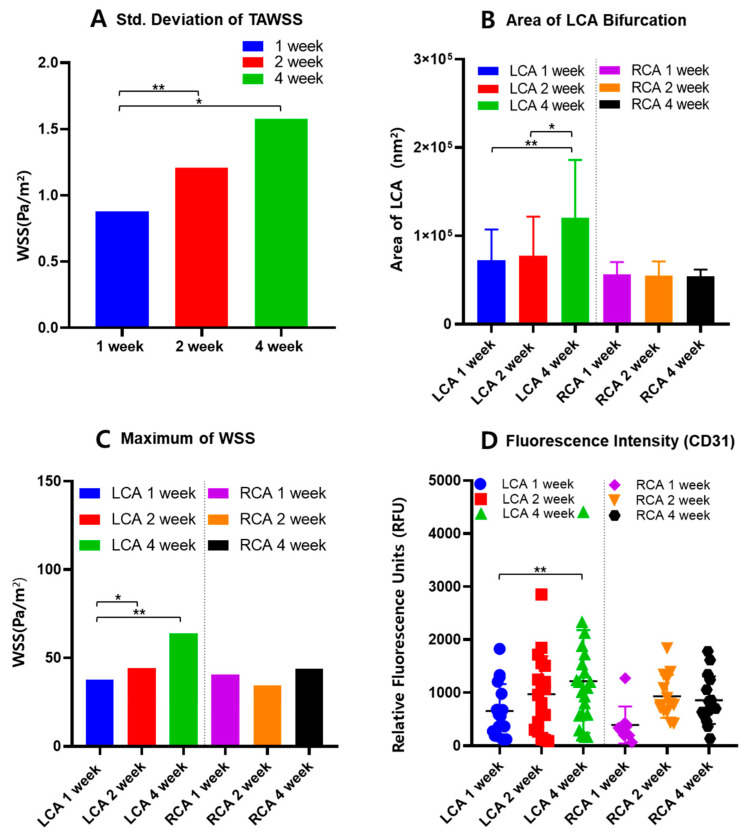
Summary of (**A**) standard deviation of TAWSS, (**B**) area, (**C**) maximum WSS, and (**D**) fluorescence intensity for LCA and RCA (*p* value < 0.01 ** and <0.05 *).

ApoE-KO mice with partial ligation and a high-fat diet were observed at 1-week, 2-week, and 4-week intervals; the CFD model shows WSS values for each interval. High WSS was observed in the partially ligated region, specifically under the bifurcation area of the LCA, as shown in Figure 4A. However, previous studies often indicate that low WSS influences the formation of atherosclerotic plaques [3,23]. Thus, this experiment was approached from the perspective that WSS can be relative to each point of the inner vessel surface. In the development of atherosclerosis, WSS does not have a specific threshold value [25]. Low WSS is one of the contributing factors to the development of atherosclerosis, but it does not have an absolute value [26]. Therefore, the WSS values at each point on the inner surface of the vessel obtained through CFD can be observed using standard deviation, which allows for the relative comparison of WSS. Atherosclerotic lesions can be relatively identified as increasing in the graph of the area excluding the inner mass of the vessel in Figure 5B, as well as in the fluorescence intensity graph in Figure 5D. As the atherosclerotic plaques progress, inflammatory biomarkers increase, leading to stronger fluorescence intensity and an increase in the entire vessel containing the atherosclerotic plaque.

Comparing the graphs of RCA and LCA for weeks 1, 2, and 4 at the same time, there was a difference in tendency between groups, as summarized in Figure 5B–D. The LCA confirms that WSS increases as weeks 1, 2, and 4 pass. In the case of the RCA, the WSS increases, but not as dramatically as in the LCA. Nevertheless, the RCA also shows an increase, which indicates that the high-fat diet fed to ApoE-KO mice caused stenosis without artificial ligation of the vessel [14]. It can be concluded that vascular stenosis can be caused by many factors, not just shear stress within the vessel [6,7].

As an analytical approach to interpreting WSS and atherosclerosis, the WSS at each point within the vessel were examined. If the deviation of WSS is large, it indicates a significant difference in WSS values within the vessel, which could predict the development of atherosclerosis. A larger standard deviation indicates a wider distribution of low and high WSS values within the vessel. The standard deviation of each group of ApoE mice is shown in Figure 5A. According to Table 1, the mean value and standard deviations for the area and maximum WSS of the LCA, standard deviation of TAWSS, and fluorescence intensity for the 1-, 2-, and 4-week mice are summarized. All values show an increasing trend over time. The standard deviation of TAWSS tended to increase with the duration of the mice survival. Therefore, the large standard deviation in TAWSS, which represents the average WSS value over one blood flow cycle, shows greater disparity between low WSS and high WSS areas. This could lead to endothelial dysfunction and low-density lipoprotein (LDL) plaque deposition. A greater disparity in WSS within the blood vessel indicates the presence of relatively low WSS [24]. This can lead to blood flow stasis, which in turn can cause atherosclerosis [6,24,27]. With standard deviation of TAWSS differences increasing at 1-week, 2-week, and 4-week intervals, the area of the LCA was compared in Figure 5B. The area of the LCA showed an increase over the weeks, confirming that low WSS influences plaque formation. The average LCA area of the 4-week mice group has increased by 66.31% from the 1-week mice group and 38.16% from 2-week mice. This significant increase shows the relationship between the relatively high or low WSS contributing to the thickened plaque within the vessel [3].

In addition, to elucidate the correlation between high WSS and inflammatory substances, the results of IF were quantified by the intensity of fluoresce. As shown in Figure 4A, the area of high WSS is under the ligation part, and, therefore, the presence of inflammatory biomarkers indicates the plaque progression. Table 2 shows that the average fluorescence intensity of CD31, an inflammatory biomarker, increased by 101.67% in the 4-week group compared to the 1-week group and 25.00% in the 2-week group. In addition, the average of the maximum WSS of the vascular model increased over time by 60.99% in the 4-week group compared to the 1-week group and 45.00% in the 2-week group. These results indicate that the presence of high WSS in the blood vessels leads to a more active inflammatory response, and the areas with relatively low WSS are continuously at an increased risk of plaque formation and atherosclerosis [24,28].

## 3. Discussion

There are many factors that contribute to the development of atherosclerosis, including wall shear stress, blood viscosity, blood pressure, and blood LDL cholesterol. In addition, high blood pressure can continuously damage the arterial walls [24]. Obesity, diabetes, and smoking can also be significant causes. However, physically low WSS in the arteries is known to be a major contributor to atherosclerotic plaque formation, so this study focused on WSS [6]. An animal model in which three of the four branches of the left common carotid artery are ligated is a classic experimental design to induce low WSS [29]. Ligation of three branches inevitably increases the area of recirculating blood flow in front of the ligation site, which increases the area with low WSS and high oscillatory shear index (OSI) [24]. This leads to plaque formation and atherosclerosis progression [6,24,27]. As atherosclerotic plaque forms, the inflammatory response can accelerate and worsen the symptoms of atherosclerosis [30,31]. In the development of atherosclerosis, low WSS can expose the arterial wall, increasing intercellular permeability and leading to the occurrence of atherosclerosis in these areas [32,33]. Additionally, it can be affected by the vasoactive substances released by endothelial cells [31]. On the other hand, high shear stress causes rapid cytoskeletal remodeling in endothelial cells and activates the signaling pathway for nitric oxide and prostacyclin, which can be protective to the atherosclerosis [34,35,36]. However, other studies suggest that high WSS affects the formation of high-risk plaques [28]. Therefore, the relationship between low WSS and high WSS in the development of stenosis along with various biomarkers and factors were examined to better understand atherosclerosis.

In this experiment, high WSS was primarily observed in ApoE-KO mice models with severe atherosclerosis through CFD simulation. When partially ligated ApoE-KO mice were subjected to a high-fat diet for 4 weeks, MRI imaging and histopathological staining techniques revealed that the severity of atherosclerotic lesions tended to increase with the duration of the survival. Regarding MRI imaging, the mice at 1 and 4 weeks showed that the blood flow was becoming increasingly obstructed. Additionally, tissues were extracted and subjected to H&E or MOVAT staining. It was observed that the vascular walls thickened, and necrotic core, lipid core, or plaque formation occurred progressively each week. However, it is difficult to attribute these findings solely to the partial ligation of the LCA and the high-fat diet, since WSS is one of the hemodynamic parameters that can be changed during the stenotic progress. Therefore, the 3D model using MRI images was constructed and then used for CFD analysis to calculate and compare the WSS in each section within the blood vessel.

The primary purpose of CFD in this study was to observe changes in WSS with the progression of atherosclerosis [8]. CFD simulations showed that WSS tended to increase over time at sites where partial ligation and high-fat diets were maintained, and higher WSS was observed at sites of branch ligation due to the formation of atherosclerotic plaques beneath these sites. Although high WSS can be a protective factor, it can also affect vulnerable plaques [28]. This is consistent with the existing understanding that high WSS may promote the expression of inflammatory factors in the chronic progression of atherosclerosis; however, high WSS alone does not fully explain plaque-induced changes in the vessel lumen, which is one of the main causes in the early stages of atherosclerosis. Analyzing only the mean of the maximum WSS may explain the risk of inflammatory factors but not the plaque formation associated with low WSS. To compensate, we analyzed the standard deviation of carotid artery thickness and TAWSS together to investigate how different patterns of WSS are associated with the progression of atherosclerosis. Standard deviation measures the variability of data, with a high standard deviation indicating greater variability in WSS. The analysis showed that the 4-week group had a high standard deviation, while the 1-week group had a relatively low standard deviation. To account for this trend, we compared the thickness of the carotid arteries and found that the area of the atherosclerotic plaque increased over time. Consequently, a higher variance indicates a higher probability of co-existing high WSS and low WSS within the blood vessel, which can indirectly be associated with the development of atherosclerosis and the formation of vulnerable atherosclerotic plaques.

As atherosclerosis progresses, the expression of various inflammatory factors and endothelial cell-related markers increases [30]. Therefore, IF staining was performed as well, targeting inflammatory biomarkers and endothelial markers such as VCAM-1, NF-κB, CD31, and α-SMA in samples from mice with advanced atherosclerosis. The results showed a higher expression pattern of these markers in samples with severe atherosclerotic plaques, which is consistent with the findings of previous studies [25]. Additionally, the fluorescence intensity from the IF images was quantified and represented. These values were then compared with the maximum WSS to observe the trend. Higher WSS was associated with increased fluorescence intensity. Therefore, it was observed that various inflammatory markers tend to aggregate due to the high WSS caused by strong pressure within the narrowed blood vessels where atherosclerosis has developed [3].

However, a limitation of this experiment is that it did not include factors other than shear stress in the vessel wall. Although atherosclerosis mainly developed in the ligated left carotid artery, atherosclerosis was also found in the un-ligated right common carotid artery, showing that shear stress in the vessel wall is not the only factor in the development of atherosclerosis [6,7]. Atherosclerosis is caused by a combination of factors, including blood viscosity, blood pressure, and structural problems in the blood vessels. However, our experiments were designed to induce low wall shear, so the focus of the study is on wall shear, which needs further study in combination with other factors [29]. Also, we used surgical methods and dietary interventions to create a model of acute atherosclerosis. The observation was conducted over a short period of 1, 2, and 4 weeks, dividing the subjects into small groups. Therefore, while only the tendency showing an increase in atherosclerotic plaques with the increase in TAWSS deviation, or the fluorescence intensity at the bifurcation where maximum WSS occurs, was confirmed, other variables related to the hemodynamics or vascular morphology of each mouse were not considered. Since there were cases where severe plaque formation occurred in mice at 1 week, and cases where significant atherosclerosis did not develop in mice at 4 weeks, these results might be due to individual differences. Therefore, to investigate the correlation in much more detail, a longer duration when creating the atherosclerosis model might be required to obtain a clearer tendency. Moreover, further research on the proximal part of the carotid artery ligation is needed. Ligation of three of the top four branches of the LCA results in stagnant blood flow and increased pressure loading proximal to the ligation [29]. This stagnation of blood flow leads to an enlargement of the recirculating region of blood flow and an increase in the low WSS and OSI regions, which are commonly believed to coincide with plaque-producing regions, which may lead to the progression of atherosclerosis [6,24,27,29]. Finally, the 3D remodeling of the vessel did not include the STA, ECA, ICA, and OA upper part of ligation, which means the shear stress effects to those parts were not considered. Consequently, accurate comparisons of low WSS and plaque formation areas might not be accomplished. Regardless of individual differences and 3D remodeling limitation, the comparison of the standard deviation of TAWSS to the area of atherosclerosis was a new approach to understanding the relationship between WSS and atherosclerotic plaque. This method can provide a new analytical perspective.

## 4. Experimental Method

### 4.1. Development of an Atherosclerosis Mouse Model

Eight-week-old C57BL/6 ApoE -/- male mice from Jackson Laboratory were used as an atherosclerosis model. For the partial ligation of the carotid artery (Figure 1A), the mice were placed under respiratory anesthesia with 2.0% isoflurane [12]. The external carotid artery (ECA), internal carotid artery (ICA), and occipital artery (OA), which branch from the LCA, were ligated, while the superior thyroid artery (STA) remained open [37]. The mice were then separated into three groups for the development of atherosclerosis: 1 week, 2 weeks, and 4 weeks. All mice were fed a high-fat diet (VHFD 60 kcal% fat #D12492, Research Diets) after the partial ligation of the LCA. MRI imaging was performed before sacrifice for blood flow measurement. All animal experiments were conducted in accordance with the guidelines and regulations set forth by the Asan Medical Center Institutional Animal Care and Use Committee (IACUC no. 2020-02-153)

### 4.2. Blood Flow Measurement through MRI

A time-of-flight (TOF) mode MRI was conducted on mice after partial ligation surgery and before their scarification (Figure 1B). This process was performed under respiratory anesthesia with 2.0% isoflurane at room temperature (24 °C). The 9.4 T/160 mm animal MR system (Agilent Technologies, Santa Clara, CA, USA) was used to obtain MRI images depicting blood flow. A 64 mm transmit/receive volume coil was used, and 16 cross-sectional slices were acquired [38].

### 4.3. Histological and Immunofluorescence Evaluation

For H&E staining and MOVAT (Figure 1C), the samples were made into paraffin blocks and cross sectioned. Both staining methods were followed using procedures from previous studies [37]. For immunofluorescence (IF) evaluation, inflammatory biomarkers NF-κB (Anti-NF-κB p65, Abcam, Cambridge, UK), CD31, VCAM-1 (VCAM-1 Monoclonal Antibody, Invitrogen, Waltham, MA, USA), and α-SMA (Abcam ab5693) were chosen. The samples prepared in paraffin blocks were deparaffinized in xylene (Junsei Chemical, Tokyo, Japan). In accordance with the primary antibody, secondary antibodies Alexa Fluor 488 (Invitrogen) and Alexa Fluor 555 (Invitrogen) were used for fluorescence tagging. DAPI was applied before fluorescent imaging. A confocal laser scanning microscope (LSM780; Carl Zeiss, Oberkochen, Germany) was used for fluorescent image acquisition.

### 4.4. Carotid Artery 3D Reconstruction

#### 4.4.1. Segmentation of Inner Vessel Cross-Section

Cross-sectional images of H&E-stained carotid artery vessels were collected to refine the inputs and outputs of the 3D model. These high-resolution images were extracted using Slide Viewer v2.7, a digital slide viewing software that standardizes the scale of all vessel cross-sectional images. The extracted images were then imported into the Segment Anything Model (SAM) API developed by Meta AI via the Python library. This API utilizes the SAM model of ViT-H to extract masks corresponding to the inner cross-sections of the vessels. These masks are used as input and output surfaces of blood flow when building the 3D model of the vessel.

#### 4.4.2. 3D Modeling

To determine the WSS applied to the vessel wall, a structured simulation approach was employed, beginning with the modeling of the left carotid artery 3D structure using MRI data (Figure 1D). The segmentation was performed using ITK-SNAP v4.0.1, a software tool designed for this purpose, allowing for accurate reconstruction of the artery’s geometric shape. A 3D model of the blood vessels was created by establishing the baseline of the curvature and thickness of the blood vessels and connecting the post-processed cross sections to the beginning and end of the blood vessels. After obtaining the ratio of the actual length of the left carotid artery specimen to the Blender modeling scale, we created a 3D model based on the ratio of all the samples. This process ensured that the detailed anatomical features were preserved and accurately represented.

### 4.5. Simulation

#### 4.5.1. Blood Vessel and Flow

For the already-formed 3D model using MRI data, the junction of the left common carotid artery and the aorta was set as the inlet of the fluid, and the area just before the division of the upper branches of the LCA was set as the outlet. The vessel wall was set as a rigid body, and the blood flow was set as laminar [39]. We set the fluid to a non-Newtonian fluid and the transport model to Bird–Carreau. The blood flow was given a velocity boundary condition based on the heart pulse, and three pulses were simulated over 2.7 s [25]. These simulations were executed using SimFlow v5.0 based on OpenFOAM v2212, which allowed us to simulate fluid dynamics. Post-processing of the simulation results was conducted using ParaView v5.12.0. This software enabled the creation of detailed visualizations of the blood flow patterns and the WSS distribution along the vessel wall, providing valuable insights into the hemodynamic environment within the carotid artery.

#### 4.5.2. Statistical Analysis

Finally, the simulation data were analyzed using Prism v8, a statistical software tool. This step involved creating graphs and charts to quantitatively represent the WSS values, helping to identify key trends and patterns that contribute to a better understanding of the impact of hemodynamic forces on the vessel wall. The *p*-value was <0.05, which means our results have statistical significance.

## 5. Conclusions

Atherosclerosis is an inflammatory response due to plaque formation within arteries, leading to ischemic stroke and heart disease [3]. It is influenced by factors like hyper-lipidemia, hypertension, obesity, diabetes, smoking, and WSS. Understanding its mechanisms is crucial for prevention and treatment of atherosclerosis. CFD simulates WSS and hemodynamic changes for analysis of the correlation between contributing factors [8]. In this study, ApoE-KO mice subjected to partial ligation and a high-fat diet at 1-week, 2-week, and 4-week intervals were used as an atherosclerosis model. CFD analysis was conducted, and carotid artery blood flow reconstructed via MRI was compared to inflammatory factors and pathological staining. This analytical approach compared the standard deviation of WSS at each point within the vessel wall with staining results and pathological features. It is found that as the interval between weeks increased, the gap between low and high WSS widened, linking the occurrence of atherosclerosis to WSS differences and vulnerable plaque formation.

## Figures and Tables

**Table 1:** Summary of LCA evaluation in area of LCA, maximum WSS, TAWSS, and fluorescence intensity.

Parameter (Mean ± SD)	1 Week (n = 16)	2 Weeks (n = 15)	4 Weeks (n = 17)
Area of LCA (nm²)	7.21 × 10⁴ ± 3.41 × 10⁴	8.68 × 10⁴ ± 4.43 × 10⁴	12.00 × 10⁴ ± 6.59 × 10⁴
Maximum WSS (Pa/m²)	39.73 ± 12.23	44.11 ± 23.68	63.96 ± 36.73
Std. Deviation of TAWSS	0.8773	1.2056	1.5787
Fluorescence Intensity (RFU)	6.00 × 10² ± 4.99 × 10²	9.68 × 10² ± 7.88 × 10²	12.1 × 10² ± 10.7 × 10²

**Table 2:** The percentage change in parameter from 1 week to 4 weeks and from 2 weeks to 4 weeks.

Parameter (Mean ± SD)	From 1 Week	From 2 Weeks
Area of LCA (nm²) (%)	66.44	38.25
Maximum WSS (Pa/m²) (%)	60.99	45.00
Std. Deviation of TAWSS (%)	79.95	30.95
Fluorescence Intensity (%)	101.67	25.00

## Data Availability

All data are contained within the article.
